# Nanopore sequencing from liquid biopsy: analysis of copy number variations from cell-free DNA of lung cancer patients

**DOI:** 10.1186/s12943-021-01327-5

**Published:** 2021-02-12

**Authors:** Filippo Martignano, Uday Munagala, Stefania Crucitta, Alessandra Mingrino, Roberto Semeraro, Marzia Del Re, Iacopo Petrini, Alberto Magi, Silvestro G. Conticello

**Affiliations:** 1 Core Research Laboratory, ISPRO, Florence, Italy; 2 Department of Medical Biotechnologies, University of Siena, Siena, Italy; 3 Department of Neuroscience, Psychology, Pharmacology and Child Health (NEUROFARBA), University of Florence, Largo Brambilla 3, 50134 Florence, Italy; 4 Unit of Clinical Pharmacology and Pharmacogenetics, Department of Clinical and Experimental Medicine, University of Pisa, Pisa, Italy; 5 Department of Experimental and Clinical Medicine, University of Florence, Florence, Italy; 6 Unit of Respiratory Medicine, Department of Critical Area and Surgical, Medical and Molecular Pathology, University Hospital of Pisa, Pisa, Italy; 7 Department of Information Engineering, University of Florence, Florence, Italy; 8 Institute of Clinical Physiology, National Research Council, Pisa, Italy

**Keywords:** Copy number aberrations. Diagnosis, Metastasis, Plasma, Third generation sequencing, cfDNA, ctDNA, Circulating tumor DNA

## Abstract

In the “precision oncology” era the characterization of tumor genetic features is a pivotal step in cancer patients’ management. Liquid biopsy approaches, such as analysis of cell-free DNA from plasma, represent a powerful and noninvasive strategy to obtain information about the genomic status of the tumor. Sequencing-based analyses of cell-free DNA, currently performed with second generation sequencers, are extremely powerful but poorly scalable and not always accessible also due to instrumentation costs. Third generation sequencing platforms, such as Nanopore sequencers, aim at overcoming these obstacles but, unfortunately, are not designed for cell-free DNA analysis.

Here we present a customized workflow to exploit low-coverage Nanopore sequencing for the detection of copy number variations from plasma of cancer patients. Whole genome molecular karyotypes of 6 lung cancer patients and 4 healthy subjects were successfully produced with as few as 2 million reads, and common lung-related copy number alterations were readily detected.

This is the first successful use of Nanopore sequencing for copy number profiling from plasma DNA. In this context, Nanopore represents a reliable alternative to Illumina sequencing, with the advantages of minute instrumentation costs and extremely short analysis time.

The availability of protocols for Nanopore-based cell-free DNA analysis will make this analysis finally accessible, exploiting the full potential of liquid biopsy both for research and clinical purposes.

**Supplementary Information:**

The online version contains supplementary material available at 10.1186/s12943-021-01327-5.

## Main text

Copy number variations (CNVs) are one of the characterizing features in many cancers: specific CNVs can define type and progression of the tumor, and are thus tightly linked to the diagnostic and prognostic process [1]. Characterization of cancer genetic features, such as CNVs, is typically done on tissue samples, either surgical resections or bioptic samples. However, the collection of tissue samples is often invasive, harmful and not repeatable [2].

On the contrary, liquid biopsy is a non-invasive approach for monitoring tumor features through analysis of body fluids. The most common approach is the analysis of cell-free DNA (cfDNA) from plasma, which can be easily collected at different time-points to follow tumor evolution, with limited harm and risks for the patient [2, 3]. However, the analysis of cfDNA is extremely challenging due to its low concentration, high fragmentation (~ 169 bp fragments) and low tumor-derived cfDNA (ctDNA) fraction (0.01–60%) [3].

Third generation sequencing approaches, such as Nanopore technology, interrogate single molecules of DNA and produce sequences much longer than those generated by second generation sequencing (SGS) methods. A sequence-dependent electrical signal is recorded as single DNA molecules pass through a pore. This allows the user to perform real- time analyses while the molecule is still being sequenced [4].

Unfortunately, as Nanopore technology is optimized for long read sequencing, its protocols are not ideal for analysis of short cfDNA fragments. Indeed, early attempts at sequencing maternal plasma cfDNA for non-invasive prenatal diagnosis resulted in unsatisfactory throughput (< 60 k reads) [5]. Thus, before Nanopore-seq potential can be exploited for liquid biopsy applications, effective and standardized workflows need to be developed.

## Results and discussion

**Fig. 1:**
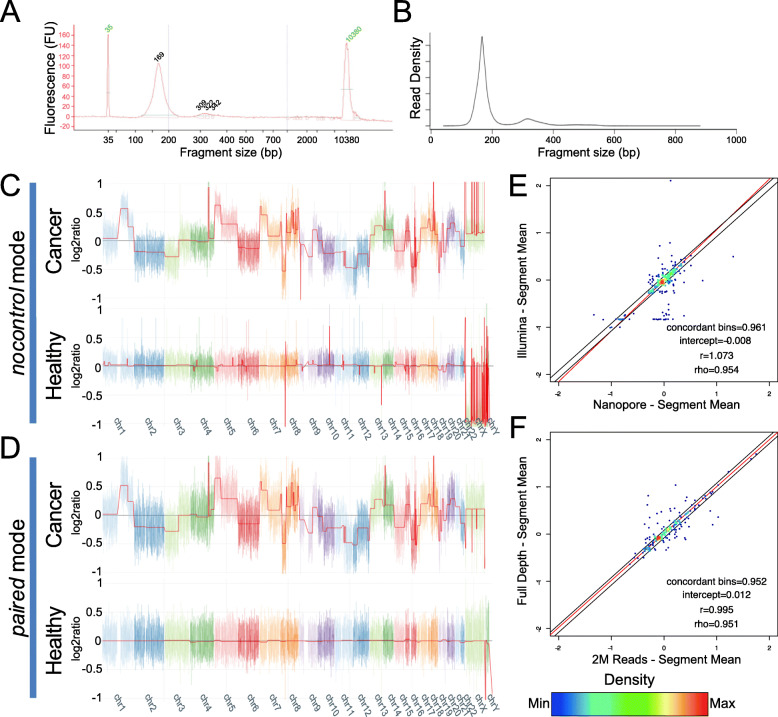
Nanopore detection of CNV from cfDNA. Fragment size distribution estimated via **a** Bioanalyzer, and from **b** Nanopore reads. NanoGLADIATOR Segmentation plots produced with NanoGLADIATOR for samples 19_1231 (cancer) and HM3 (healthy) in **c** “nocontrol” mode, and **d** “paired” mode. In “paired” mode, HF1 and HM2 have been used as controls for respectively 19_1231 and HM3. The red line indicates the segment mean (log2ratio). Each color represents a different chromosome; chromosome Y has been omitted for sample 19_123. Comparison of segmentation results: **e** Correlation of Nanopore and Illumina segment mean values (sample 19_744); **f** Comparison of Nanopore segment mean values from the full-depth BAM file and from a 2 M-reads subsampled dataset (sample 18_1130). Each genomic bin is represented as a dot, colors indicate dot density. Regression lines are shown in red. Black lines indicate the thresholds for concordant bins

We have modified Nanopore standard protocols to make them compatible with small cfDNA fragments (see Additional file 1: Methods). We sequenced cfDNA from 6 cancer patients and 5 healthy subjects, in both single-plex and multi-plex runs (S1, M1 and M2). We obtained 14,338,633, 19,610,131, and 31,582,051 raw reads from the S1, M1 and M2 runs, respectively: a remarkably higher throughput than previously reported [5] (Additional file 2: Table S1). The higher throughput is attributable to updates in the Nanopore protocol (SQK-LSK109 kit and R9.4.1 flow cells instead of SQK-MAP-005 and R7.3) and to adjustments in the clean-up beads volume to retain smaller fragments. The per-sample throughput was highly variable. Indeed, the throughput obtained for sample HF2 was insufficient. Such differences are likely to depend on variable efficiencies in the library preparation rather than in the amount of input DNA (see Additional file 1: Supplementary Results). Size distribution of the sequenced cfDNA fragments perfectly matches the fragmentation profile obtained with Agilent Bioanalyzer (Fig. 1a and b).

Molecular karyotype of 10 out of 11 samples was successfully produced using NanoGLADIATOR (“nocontrol” mode), a recently developed tool for the identification of CNVs from read counts (reported as log2ratio) across multiple consecutive genomic windows (bins) [6]. BWA-aligned BAM files were analyzed with a bin size of 100kbp, and CNVs were detected in all the tumoral samples (Fig. 1c, Additional file 3: Fig. S1).

Unexpected CNVs were present also in samples from healthy donors (Fig. 1c, Additional file 3: Fig. S2). Most of the variations observed in healthy donors are shared by at least 2 healthy subjects, suggesting that they may be errors introduced by the technique itself rather than patient-specific alterations (Additional file 3: Fig. S3). Even though it is possible that these variations represent naturally occurring polymorphisms, this is unlikely: polymorphic variations should present a discrete number of copies (1,3 or 4 copies), which is not the case, as most of these variations have weak log2ratios.

To further confirm that these CNVs were indeed artifacts, we sequenced the genomic DNA from white blood cells of one healthy control (HM1) and no CNVs were detected (Additional file 3**:** Fig. S4).

These technical artifacts can be easily filtered out by setting a threshold. On the other hand, some of these variations are very similar in terms of length and segment mean (roughly ±0.10) to those we observe in cancer samples and it could be difficult to discriminate real CNVs from these ones (Fig. 1c, Additional file 1: Supplementary Results, Additional file 3: Fig. S5). Typically, these artifacts are present in regions containing a higher number of similar sequences, e.g. the sex chromosomes (Additional file 3: Fig. S2). Alignment of short reads in such genomic regions is typically challenging and presence of these artifacts is likely due to mapping issues [7]. In order to minimize the number of artifacts, we used NanoGLADIATOR in “paired” mode, which generates segmentation results comparing test samples with a control sample. In “paired” mode, we used as control a merged BAM files from the samples of healthy donors that allowed us to decrease the log2ratio of these artifacts to ±0.04 and, consequently, to drastically increase the specificity of the analysis (Fig. 1d, Additional file 1: Supplementary Results, Additional file 2: Table S2).

We then compared the performance of Nanopore sequencing with a standard SGS approach by analyzing four of the tumoral samples through Illumina sequencing (17-24 M, 150 bp single end reads, see methods). Illumina and Nanopore results (“nocontrol” mode) were strongly correlated (*R* = 0.93–0.99, *p* < < 0.001), with concordant log2ratio values at 95–98% of the genomic bins (Fig. 1e, Additional file 2: Table S3). Using the Illumina results as true-positive dataset, the Nanopore approach, on average, resulted in 94% sensitivity, 89% specificity, 94% accuracy and 96% precision (Additional file 2: Table S4). Nanopore cfDNA results also showed a high correlation with long-read sequencing (*R* = 0.88, *p* < < 0.001), with concordant log2ratio values at 90% of the genomic bins (Additional file 3: Fig. S6, A). We also determined that our approach is capable to detect CNVs with as little as 5–10% of ctDNA fraction, similarly to what has been reported with regard to Illumina sequencing [8, 9]. To assess the performances of our approach at even lower sequencing depth, we subsampled the BAMs to 2 M raw reads: the results obtained are highly concordant with the full-depth BAMs (*R* = 0.93–0.99, *p* < < 0.001, 94–99% concordant bins, Fig. 1f, Additional file 2: Table S5). The marginal loss of performance observed is comparable to the one obtained when subsampling Illumina data (Additional file 2: Table S6).

**Fig. 2:**
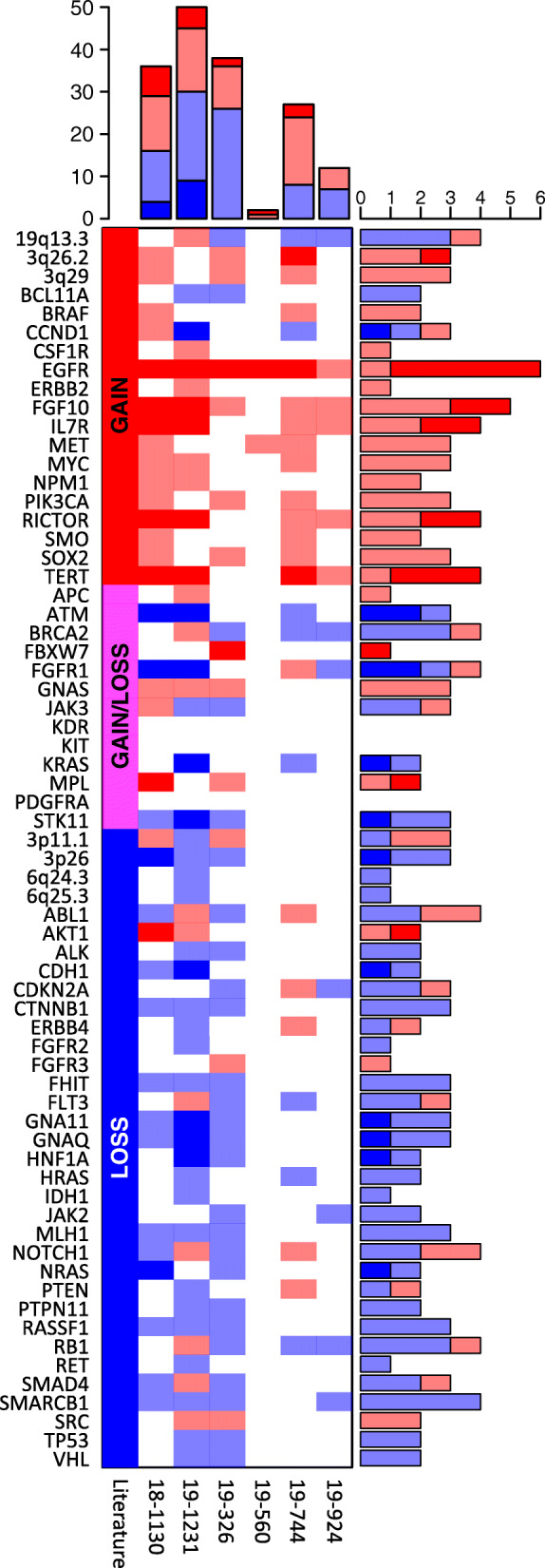
Landscape of clinically-relevant copy number variants. Copy number variants of specific genes (rows) are shown for the individual patients (columns). The shading indicates levels of amplification (red tones, 0.10–0.30, > 0.30 log2ratio) and deletion (blue tones, 0.10–0.30, > 0.30 negative log2ratio). The top and right bar plots show the number of CNVs in one patient and the number of patients with CNVs for a given gene, respectively. The expected status for a given gene based on the literature is shown in the left side

Since the ultimate aim of the analysis is to obtain information on the tumor, we next assessed the status of genes and genomic regions commonly altered in lung cancer (Fig. 2, Additional File 1: Methods). Pathogenic CNVs were readily observed, with EGFR amplification prominently present in all samples, and most of other genes altered in at least two samples. Many of these structural alterations directly affect progression of the cancer and therapeutic options. For example, RICTOR amplification identifies a subgroup of lung cancer and its presence has been linked to the response to mTOR inhibitors [10]. Similarly, MYC amplification confers resistance to pictilisib in models and PIK3CA amplification is associated with resistance to PI3K inhibition [11, 12] in mammary tumors.

Our report is the first successful attempt to obtain a CNV profile from plasma cell-free DNA of cancer patients using Nanopore technology. Our results show that Nanopore sequencing has the same performance of SGS approaches and, in terms of throughput and sequencing costs, it is comparable to an Illumina MiSeq run (V3 reagents, 22-25 M single-end reads).

MinION is the entry-level sequencer by Nanopore technology, and its cost is extremely low (~ 1000 euros) compared to SGS sequencers. Reduced overall instrumentation costs makes this approach accessible to most of the research groups, which would otherwise be forced to outsource the sequencing, or to gain access to shared sequencers, leading often to long queues and delays. Moreover, SGS is cost effective only when dealing with a large number of patients. This aspect is crucial with regards to clinical analyses, as it leads to a centralization of sequencing-based assays, which are mainly performed in big hospitals that collect samples from larger geographic areas.

On the contrary, Nanopore technology is extremely scalable, and only a modest number of patients is required in a multiplexed run, leading to short recruitment times and, consequently, faster results.

As we demonstrate that reliable results can be obtained from as few as 2 M reads. Based on the throughput obtained in our study, it should be possible to analyze up to 7–15 patients in a single run.

Since reads are stored as soon as they are produced, they can be analyzed while the experiment is still running by taking advantage of the real-time mode of NanoGLADIATOR. This feature might come useful when analyzing single samples, especially in those patients with lower fraction of ctDNA, for which a higher number of reads and, consequently, a higher resolution may be preferable. In such a context, it would be possible to inspect the CNV profile while the run is still ongoing, and stop it once the desired resolution is reached, saving the sequencing power of the flow cell, which can be washed and reused for other samples.

According to our sequencing statistics, 2 M reads are produced in less than 3 h. This means that the entire workflow -from blood withdrawal to bioinformatic analyses- can be performed in less than a working day. This is something unique to Nanopore sequencing, as SGS approaches based on sequence-by-synthesis technologies make reads available only at the end of the whole run, which can last days. We have demonstrated that Nanopore sequencing for CNV analysis of short plasmatic cfDNA is feasible. Nanopore sequencing provides several advantages over current sequencing technologies and might drive the adoption of molecular karyotyping from liquid biopsies as a tool for cancer monitoring in clinical settings. The applications of this approach are not limited to cancer and can be technically extended to other liquid biopsy-based fields such as noninvasive prenatal diagnosis.

## Supplementary Information

**Additional file 1: Methods and supplementary results.** Pdf file including more detailed information on methods and results.

**Additional file 2: Supplementary tables.** Spreadsheet file including analyzed data and statistics. **Table S1.** Case series and run statistics**. Table S2.** Performance of NanoGLADIATOR pipeline in “nocontrol” and “paired” mode**. Table S3**. Correlation of Illumina and Nanopore results. **Table S4.** Sensitivity, Specificity, Accuracy and Precision test of Nanopore approach. **Table S5.** Correlation of Nanopore results: subsampled BAMs (2 M reads) Vs full BAMs (“nocontrol” and “paired” mode)**. Table S6.** Correlation of Illumina results: paired-end Vs single-end, and subsampled BAMs (2 M reads) Vs full BAMs**. Table S7**. Genes and genomic regions CNV results**. Table S8.** CNV detection performance at different tumor fractions.

**Additional file 3: Supplementary Figures. Fig. S1**. Segmentation results of cancer patients, “nocontrol” mode. **Fig. S2**. Segmentation results of healthy subjects, “nocontrol” mode. **Fig. S3**. Technical artifacts in healthy samples. Venn diagram reporting recurring genomic bins with altered log2ratio in healthy samples. **Fig. S4**. Segmentation results of HM1 white blood cells. **Fig. S5**. Segment mean and segment length of Nanopore results. Correlation of segment mean and length in nocontrol **(A)** and paired mode **(B)**. Every dot represents a segment. Segment mean is reported on the x-axis and segment length (number of bins per segment) on the y axis. Vertical lines indicate the threshold used to discriminate artifacts from CNVs (log ratio ± 0.04). The lower range of the segments is shown in the lower plot for each sample. **Fig. S6**. Correlation of short- and long-read sequencing results. **(A)** Correlation plot of short (sheared DNA) and long (non-sheared DNA) sequencing. Each genomic bin is represented as a dot, colors indicate dot density. Regression lines are shown in red. Black lines indicate the thresholds for concordant bins. **(B)** Fragment length distribution of HEK_sheared sample obtained from read length. Vertical lines indicates 160 and 320 bp length. **Fig. S7**. Segmentation results of cancer patients, “paired” mode.

## Data Availability

The dataset supporting the conclusions of this article is available in the EGA repository (https://ega-archive.org/, project # EGAD00001006888).

## Abbreviations

cfDNA: Cell-free DNA
CNV: Copy number variation
ctDNA: Tumor-derived cfDNA
SGS: Second generation sequencing
