# Supplementary material for: Nanopore sequencing from liquid biopsy: analysis of copy number variations from cell-free DNA of lung cancer patients
Source: Mol Cancer. 2021 Feb 12;20:32. doi: 10.1186/s12943-021-01327-5 (PMC7881593; doi:10.1186/s12943-021-01327-5)
Supplement: Supplementary file 1 — Additional file 1: Methods and supplementary results. Pdf file including more detailed information on methods and results. [file 12943_2021_1327_MOESM1_ESM.docx]

**Methods**

**Sample collection and cfDNA isolation**

Blood from 5 unrelated healthy donors and 6 unrelated metastatic Non Small Cell Lung Cancer patients was collected in EDTA vacuum tubes. Blood samples were centrifuged at 1600g x 10”, and plasma was carefully collected with a pipet without disturbing sedimented blood cells. cfDNA was extracted from 4ml of plasma using QIAamp Circulating Nucleic Acid Kit (QIAGEN, 55114), it was quantified via Qubit Fluorometer (Thermo Fisher Scientific, dsDNA HS assay kit, Q32851), and its fragmentation pattern was obtained via Agilent 2100 Bioanalyzer (Agilent, High Sensitivity DNA kit, 5067-4626). Extracted cfDNA was stored at -80° C.

**Preparation of genomic DNA and simulated cfDNA.**

Genomic DNA was extracted from 200 ul of White Blood Cells from sample HM1 using QIAamp DNA Blood Mini Kit (QIAGEN, 51104).

Genomic DNA from HEK293T cells was extracted using the Wizard Genomic DNA Purification Kit (Promega, A1125). HEK293T cells were cultured at 37 °C, 5% CO_2_, in Dulbecco’s modified eagle medium supplemented with 10% fetal bovine serum, 2 mM L-glutamine, and 1 mM penicillin/streptomycin.

Genomic DNA was sheared by sonication using a Sonopuls GM3200 (Bandelin), 100 cycles (30” on;30” off) at 4°C. In order to purify and retain only the smaller fragments, we optimized the clean-up protocol with AMPure beads. To remove high molecular weight fragments the DNA sample with 1.2 volumes of beads solution was incubated on a hula mixer for 10 min. After segregation of the magnetic beads, supernatant was recovered and 3 volumes of beads solution were added, after washing twice with 70% EtOH, the beads were dried for no longer than 30 s before eluting small molecular weight DNA in 50 µl Tris–HCl pH 8.0. Fragment distribution was first assessed by agarose gel electrophoresis.

**Nanopore library preparation and analysis**

For library preparation of the genomic DNA from HEK293T and White Blood Cells, the SQK-LSK109 protocol were used following manufacturer’s instructions.

For library preparation of cfDNA samples (including HEK293T simulated cfDNA), the EXP-NBD104 and SQK-LSK109 protocols were used. In contrast to what suggested by the protocol, the bead/sample ratio of AMPure XP beads (Beckman Coulter, A63880) was increased to 1.8x in all clean-up steps.

All the other steps were performed following the manufacturer’s instructions.

The SQK-LSK109 protocol was used for the run S1. In the case of the multiplex runs M1 and M2, 25ul of each barcoded sample were pooled together before adapter ligation. The pool was then cleaned-up using 2.5X AMPure XP beads.

S1, M1 and M2 runs were performed using FLO-MIN106 (R9.4) flow cells on a GridION sequencer. FASTQ files were generated via real-time high-accuracy basecalling during the run, and Porechop (https://github.com/rrwick/Porechop) was used to de-multiplex FASTQ files of multiplex runs (M1, M2), and to trim adapters of all the runs.

Minimap2 (with *-ax map-ont* flags) (1) and BWA mem (with *-x ont2d* flags) (2) were used to align raw reads, using the human_g1k_v37_decoy as reference genome.

BWA aligned bams were used for subsequent analyses due to higher percentage of mapped reads compared to Minimap2 (see **Supplementary Results, Additional file 2: Table S1**).

The CIGAR field of aligned BAMs was used to determine fragment length of sequenced cfDNA (**Figure 1B**).

NanoGLADIATOR was used to generate molecular karyotypes of BWA aligned BAMs with a bin size of 100kb (3).

For “paired” mode analysis, HF1 was used as a control for female patients, and BAMs from HM2 and HM3 were merged and used as control (Healthy_Males_Pool, HMP) for male patients (see supplementary results).

Additional details on patients features, library preparation and run statistics are summarized in **Additional file 1: Table S1**.

**Illumina library preparation and analysis**

Illumina libraries for samples 19_924, 19_744, 19_1231 and 18_1130 were prepared from 15ng of input DNA, using Ovation Ultralow V2 DNA-seq Library Preparation Kit (NUGEN, 0344NB-A01), sequencing runs (150bp, paired end) were performed on a NovaSeq 6000 sequencer (Illumina).

Only R1 reads were used for CNV analysis treating them as the product of a single end sequencing experiment, in order to simplify subsequent steps such as subsampling and comparison with Nanopore results. This strategy doesn’t introduce any methodological bias, since Illumina single-end and paired-end CNV results are highly correlated (**Additional file 1:** **Table S6**).

FASTQ files were aligned with BWA mem using human_g1k_v37_decoy as reference genome.

XCAVATOR was used to generate molecular karyotypes of BWA aligned BAMs with a bin size of 100kb (4).

**Segmentation comparison**

Custom R scripts were used to compare segmentation results:

When comparing two experiments, the “segment mean” value of each of the 100kb bins was correlated (corr.test function, R base package, method=”spearman”).

To determine the percentage of genomic positions with concordant copy number status, we considered two bins as “concordant” if their segment mean differs by ±0.08.

Chromosome Y bins were ignored when analyzing female patients.

When comparing Illumina and Nanopore results, even if the bin size used was the same, the starting positions of the bins slightly differs among the two pipelines; an XCAVATOR bin is considered corresponding to a NanoGLADIATOR bin if its starting position falls between the starting and the end position of the NanoGLADIATOR bin. Only NanoGLADIATOR bins for which it was possible to identify a corresponding XCAVATOR bin were considered for subsequent analysis.

**Gene/region CNV detection**

The log2ratio value for each gene/region was calculated as the median of the segment mean values of the bins whose position falls between the start and stop of the gene/region. Using read-count based methods such as NanoGLADIATOR, the presence of large and numerous CNVs can complicate the definition of the expected read-count for diploidy. Taking in account this limitation, we used a stricter log2ratio thresholds to avoid false positives:

< -0.30: Strong Loss

≥ -0.30 & <-0.10: Weak Loss

> 0.10 & < 0.30: Weak Gain

> 0.30: Strong Gain

Genes and genomic regions were chosen based on previous reports (5-11).

Log2Ratio values and genes’ characteristics are summarized in **Additional File 2: Table S7**.

**Supplementary Results**

**Choice of alignment tool**

Alignments were performed with both Burrows Wheeler Aligner (BWA) and Minimap2 (see methods). The average percentage of uniquely mapped reads was 98.5% and 85.6%, respectively (**Additional file 2: Table S1**). While Minimap2 is usually recommended for alignment of long Nanopore reads, according to our results BWA is preferable for cfDNA-derived data, probably due to the shorter length of cfDNA fragments.

**Throughput variability**

To assess the effects of input DNA on per-sample throughput, we performed library preparation of samples HM2, HM1 and HF1 with respectively 15, 30 and 60 ng of DNA; however, the amount of reads produced was very consistent among the three samples (~3M reads, **Additional file 2: Table S1**), suggesting that input DNA has a low impact on the final throughput.

For the run M2, we quantified eluted DNA after each clean-up step via Qubit Fluorometer: Since DNA concentration highly correlates with read yield, differences in per-sample yields are likely attributable to a different efficiency of library preparation steps rather than amount of input DNA.

Nanopore protocols suggest pooling equimolar quantities of barcoded samples prior to adapter ligation to avoid differences in per-sample throughput. However, in order to avoid any waste of DNA and aiming at obtaining the maximum amount of reads from a single flow-cell, we loaded the entire barcoded sample for each patient, which may explain the observed variability.

Unexpectedly, the relative-throughput (sample reads/total run reads) of cancer patients is remarkably higher compared to healthy subjects (**Additional file 2: Table S1**). Since there were no differences in input DNA, and per-sample throughput depends mainly on library preparation efficiency, it is possible that the presence of ctDNA positively affects library preparation efficiency; however, the biological aspects of this behavior are not clear and should be further investigated.

**Artifact filtering using NanoGLADIATOR in “paired” mode**

We used NanoGLADIATOR in “paired” mode with the aim of correcting eventual method-specific artifacts: we tested it on healthy male subjects, using each sample as both case and control, in any possible combination. Using this strategy, we were able to remove 82-100% of false positive bins in healthy male samples (segment mean threshold ≥ 0.04, or ≤ -0.04) (**Additional file 2: Table S2, Additional file 3: Figure S5**). Moreover, removal of HM1 data from this pairwise comparison reduced the number of false positive bins by 100% (**Additional file 2: Table S2**), suggesting that this sample might be enriched in sample-specific artifacts. Hence, HM1 was not used as a control in subsequent “paired” analyses to avoid introduction of biases. HM2 and HM3 BAM files were merged and the resulting BAM (HMP) was used as control for male patients, while HF1 was used as control for female patients (**Additional file 3: Figure S7**).

This approach doesn’t negatively affect the performance of the analysis, as the number of copy-number altered bins is reduced by less than 5% in most of the tumoral samples and increases by 29% in sample 19_744; sample 19_560 is the only exception, with a reduction of roughly ~40% (**Additional file 2: Table S2**). 19_560 shows the lowest number of altered bins and the lowest segment mean standard deviation (calculated on autosomes) (**Additional file 2: Table S2**). A lack of clonal CNVs in the tumor, or a lower concentration of ctDNA fragments among the overall cfDNA population can explain these observations; it is therefore not surprising to observe an “healthy-like” genotype, with false positives representing a large part of the detected CNVs.

Using NanoGLADIATOR in “paired” mode allows to set a very strict log2ratio threshold (±0.04) to discriminate technical artifacts from real CNV, drastically increasing the performance of the approach in terms of sensitivity/specificity (**Figure 1, Additional file 2: Table S2**).

**Comparison between short-read and long-read sequencing**

As CNVs called using Nanopore sequencing were matching those called using Illumina, we wanted to assess the concordance between Nanopore sequencing based on short-reads -cfDNA from liquid biopsy- and long-reads -whole genome from white blood cells. While the expected lack of CNVs in the HM1 samples prevented us to assess the concordance, we were prevented to compare also CNVs found in the cfDNA with those found in the primary or metastatic tumors: cfDNA comes from a mixture of different tumoral clones while tissue-based analysis is subjected to sampling bias that would make the comparison unfeasible. We therefore resorted to analyze CNV heterogeneity in a genetically unstable cell line such as HEK293T cells.

To this aim we have prepared and sequenced a “simulated” cfDNA sample (~6 Million reads), obtained by shearing HEK293T genomic DNA and size selecting it to enrich 160bp fragments (**Additional file 3: Figure S6, B**). The results were compared with a long-read sequencing of the same genomic DNA prior shearing (~1.2 Million reads).

CNVs called from the two sequencing runs showed a high correlation (R = 0.88, p << 0.001) and a high percentage of concordant bins (90%) (**Additional file 3: Figure S6, A**), suggesting that CNVs obtained from cfDNA resemble the actual genomic status of the original tumor(s).

**Detection limits of Nanopore cfDNA analysis**

It is important to understand the minimal detectable fraction of a clonal CNV in the whole cfDNA. Given the challenge to estimate the actual ctDNA fraction from in the patient samples, our cancer sequencing data would not be reliable as "true positives" to assess the detection limits of the approach. We thus generated a simulated true-positive standard using the data from the healthy subjects. Sequencing reads from the HM2 sample were used to provide the baseline tumor genome containing 3 artificial CNVs: a deletion (obtained by removing half of the reads in region chr7:550,000-57,000,000), a 3-copies amplification (obtained by adding HF1 reads from region chr7:100,000,000-150,000,000) and a 4-copies amplification (obtained by adding HF1 and HM1 reads from region chr7:63,000,000-100,000,000). This artificial genome was used to spike the sequencing reads from one healthy subject (HM3), which provided the baseline diploid cfDNA.We have used different ratios of simulated ctDNA and cfDNA in order to obtain samples with different ctDNA fractions (50%,40%,30%,20%,10%,5%,2%,1%).

All the simulated CNVs were detected with as few as 5% ctDNA fraction (**Additional file 2: Table S8**). Notably, the Log2Ratio values of 3- and 4-copies amplifications were very similar when a 5% ctDNA fraction was used. This suggests that a ctDNA fraction of at least 10% is necessary to discriminate low-copies and high-copies amplifications.

**Supplementary Bibliography**

1. Li H. Minimap2: pairwise alignment for nucleotide sequences. Bioinformatics (Oxford, England). 2018;34(18):3094-100.

2. Li H, Durbin R. Fast and accurate short read alignment with Burrows-Wheeler transform. Bioinformatics (Oxford, England). 2009;25(14):1754-60.

3. Magi A, Bolognini D, Bartalucci N, Mingrino A, Semeraro R, Giovannini L, et al. Nano-GLADIATOR: real-time detection of copy number alterations from nanopore sequencing data. Bioinformatics (Oxford, England). 2019.

4. Magi A, Pippucci T, Sidore C. XCAVATOR: accurate detection and genotyping of copy number variants from second and third generation whole-genome sequencing experiments. BMC genomics. 2017;18(1):747.

5. Liao Y, Ma Z, Zhang Y, Li D, Lv D, Chen Z, et al. Targeted deep sequencing from multiple sources demonstrates increased NOTCH1 alterations in lung cancer patient plasma. Cancer medicine. 2019;8(12):5673-86.

6. Peng H, Lu L, Zhou Z, Liu J, Zhang D, Nan K, et al. CNV Detection from Circulating Tumor DNA in Late Stage Non-Small Cell Lung Cancer Patients. Genes. 2019;10(11).

7. Chen X, Chang CW, Spoerke JM, Yoh KE, Kapoor V, Baudo C, et al. Low-pass Whole-genome Sequencing of Circulating Cell-free DNA Demonstrates Dynamic Changes in Genomic Copy Number in a Squamous Lung Cancer Clinical Cohort. Clinical cancer research : an official journal of the American Association for Cancer Research. 2019;25(7):2254-63.

8. Vanhecke E, Valent A, Tang X, Vielh P, Friboulet L, Tang T, et al. 19q13-ERCC1 gene copy number increase in non--small-cell lung cancer. Clinical lung cancer. 2013;14(5):549-57.

9. Sakre N, Wildey G, Behtaj M, Kresak A, Yang M, Fu P, et al. RICTOR amplification identifies a subgroup in small cell lung cancer and predicts response to drugs targeting mTOR. Oncotarget. 2017;8(4):5992-6002.

10. Bowcock AM. DNA copy number changes as diagnostic tools for lung cancer. Thorax. 2014;69(5):496.

11. Du M, Thompson J, Fisher H, Zhang P, Huang CC, Wang L. Genomic alterations of plasma cell-free DNAs in small cell lung cancer and their clinical relevance. Lung cancer (Amsterdam, Netherlands). 2018;120:113-21.
