# Supplementary material for: Nanopore sequencing from liquid biopsy: analysis of copy number variations from cell-free DNA of lung cancer patients
Source: Mol Cancer. 2021 Feb 12;20:32. doi: 10.1186/s12943-021-01327-5 (PMC7881593; doi:10.1186/s12943-021-01327-5)
Supplement: Supplementary file 3 — Additional file 3: Supplementary Figures. Fig. S1. Segmentation results of cancer patients, “nocontrol” mode. Fig. S2. Segmentation results of healthy subjects, “nocontrol” mode. Fig. S3. Technical artifacts in healthy samples. Venn diagram reporting recurring genomic bins with altered log2ratio in healthy samples. Fig. S4. Segmentation results of HM1 white blood cells. Fig. S5. Segment mean and segment length of Nanopore results. Correlation of segment mean and length in nocontrol (A) and paired mode (B). Every dot represents a segment. Segment mean is reported on the x-axis and segment length (number of bins per segment) on the y axis. Vertical lines indicate the threshold used to discriminate artifacts from CNVs (log ratio ± 0.04). The lower range of the segments is shown in the lower plot for each sample. Fig. S6. Correlation of short- and long-read sequencing results. (A) Correlation plot of short (sheared DNA) and long (non-sheared DNA) sequencing. Each genomic bin is represented as a dot, colors indicate dot density. Regression lines are shown in red. Black lines indicate the thresholds for concordant bins. (B) Fragment length distribution of HEK_sheared sample obtained from read length. Vertical lines indicates 160 and 320 bp length. Fig. S7. Segmentation results of cancer patients, “paired” mode. [file 12943_2021_1327_MOESM3_ESM.pdf]

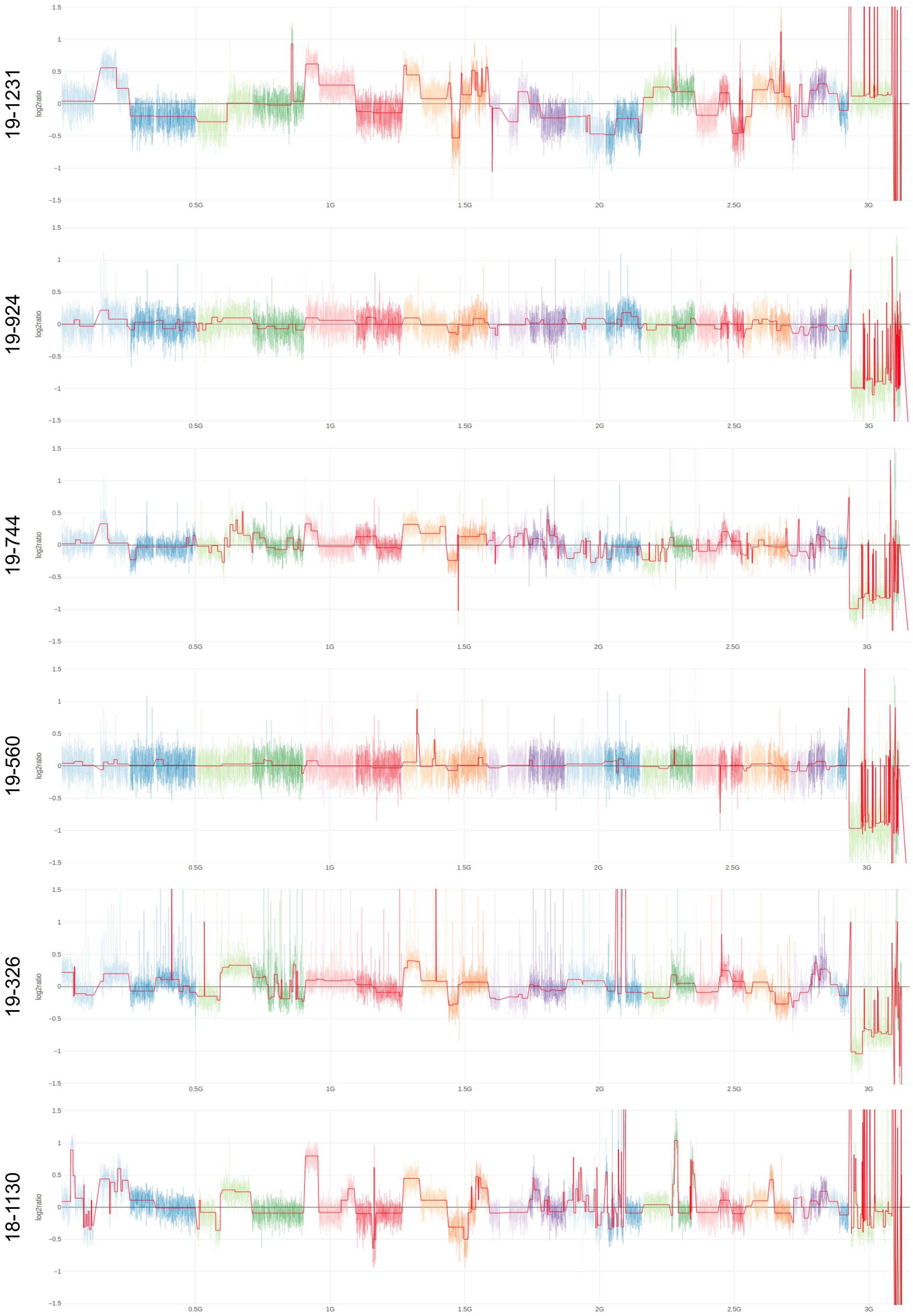

Figure S1. Segmentation results of cancer patients, *nocontrol* mode

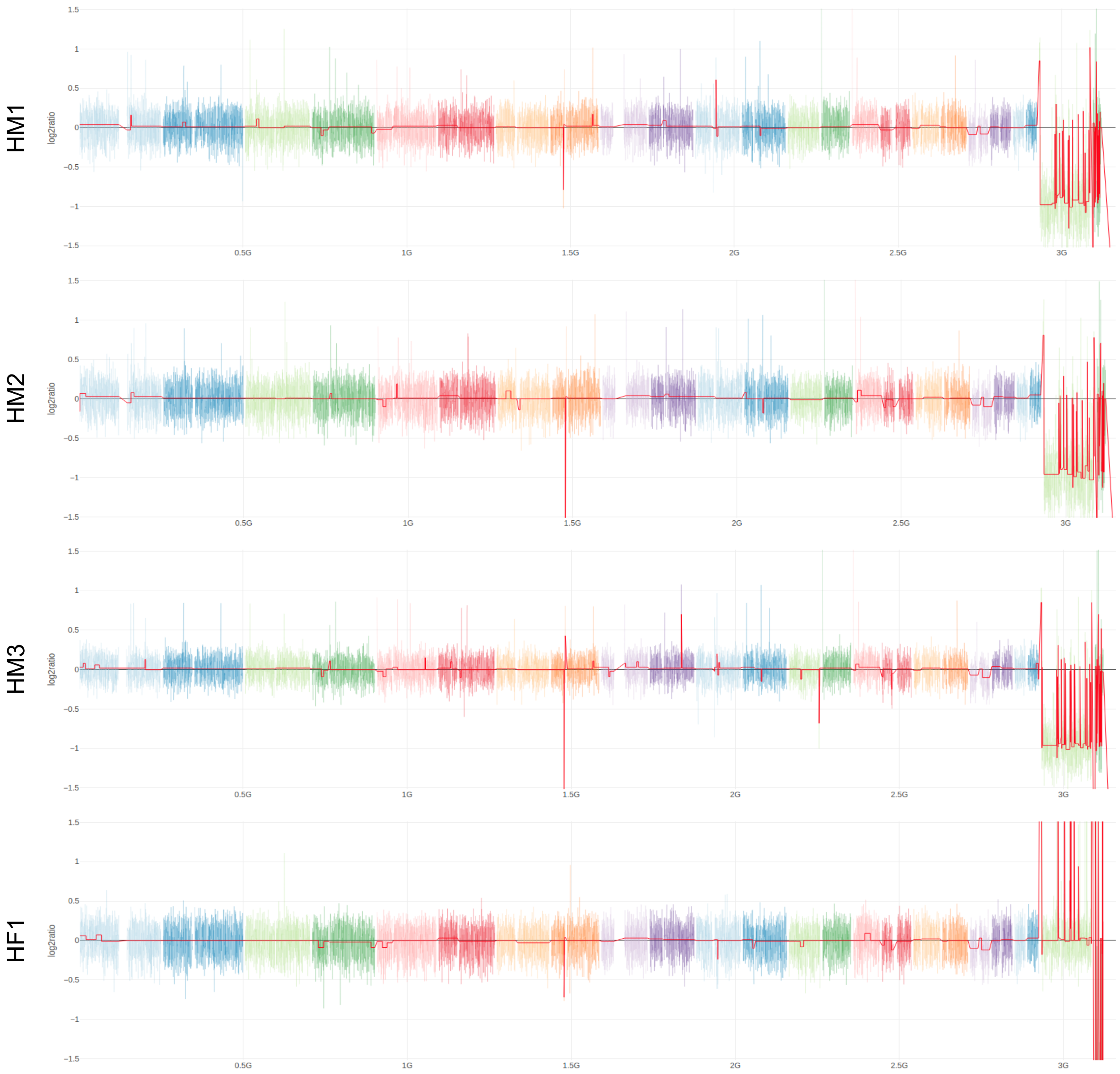

Figure S2. Segmentation results of healthy subjects, *nocontrol* mode

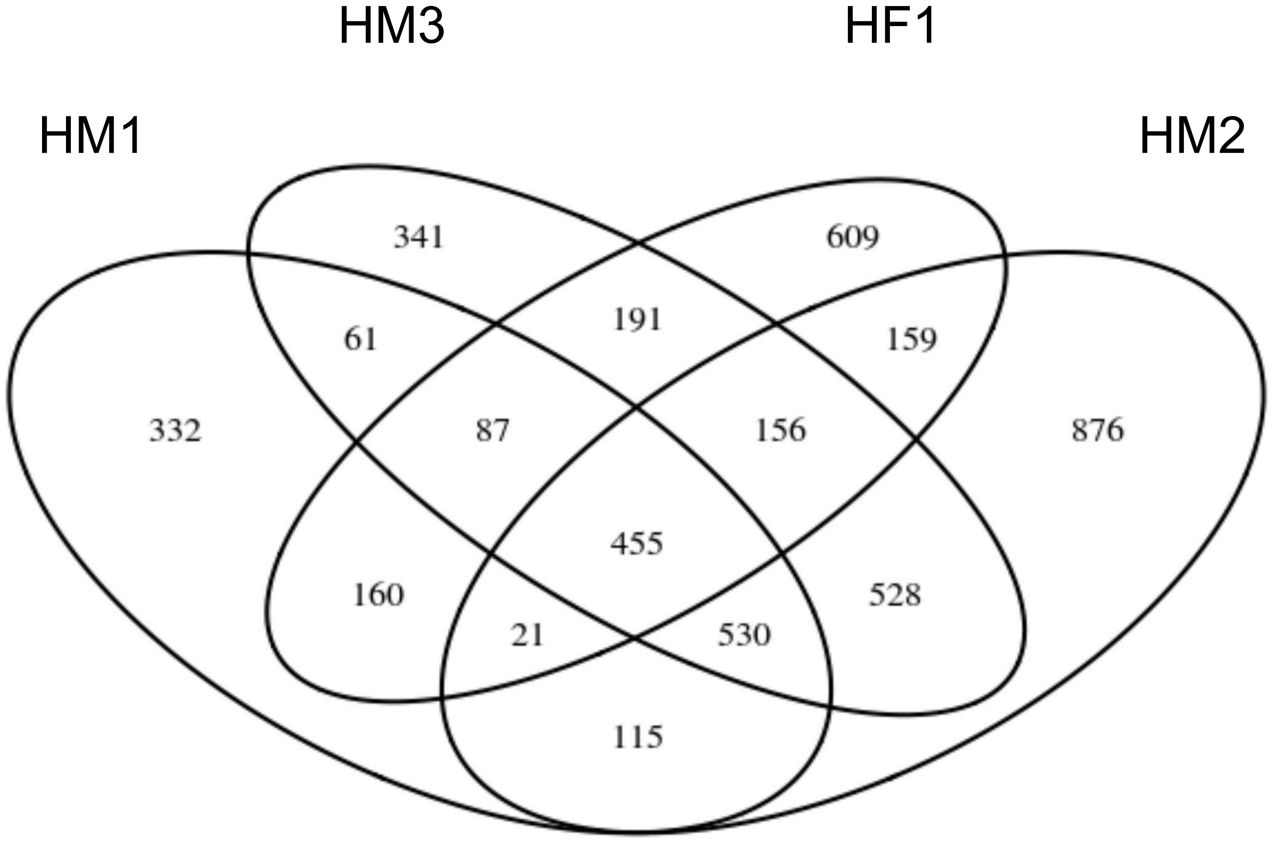

Figure S3. Technical artifacts in healthy samples
Venn diagram reporting recurring genomic bins with altered log2ratio in healthy samples.

HM1 White Blood Cells

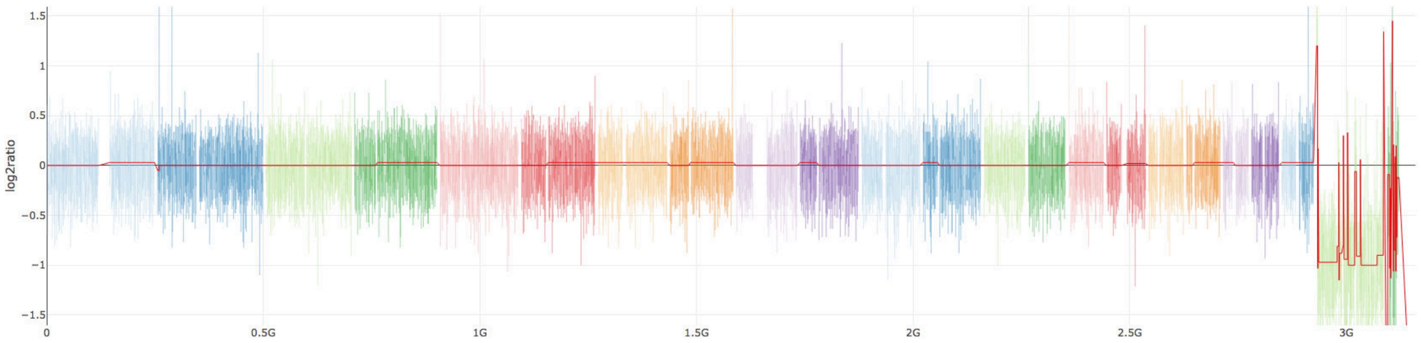

Figure S4. Segmentation results of HM1 white blood cells

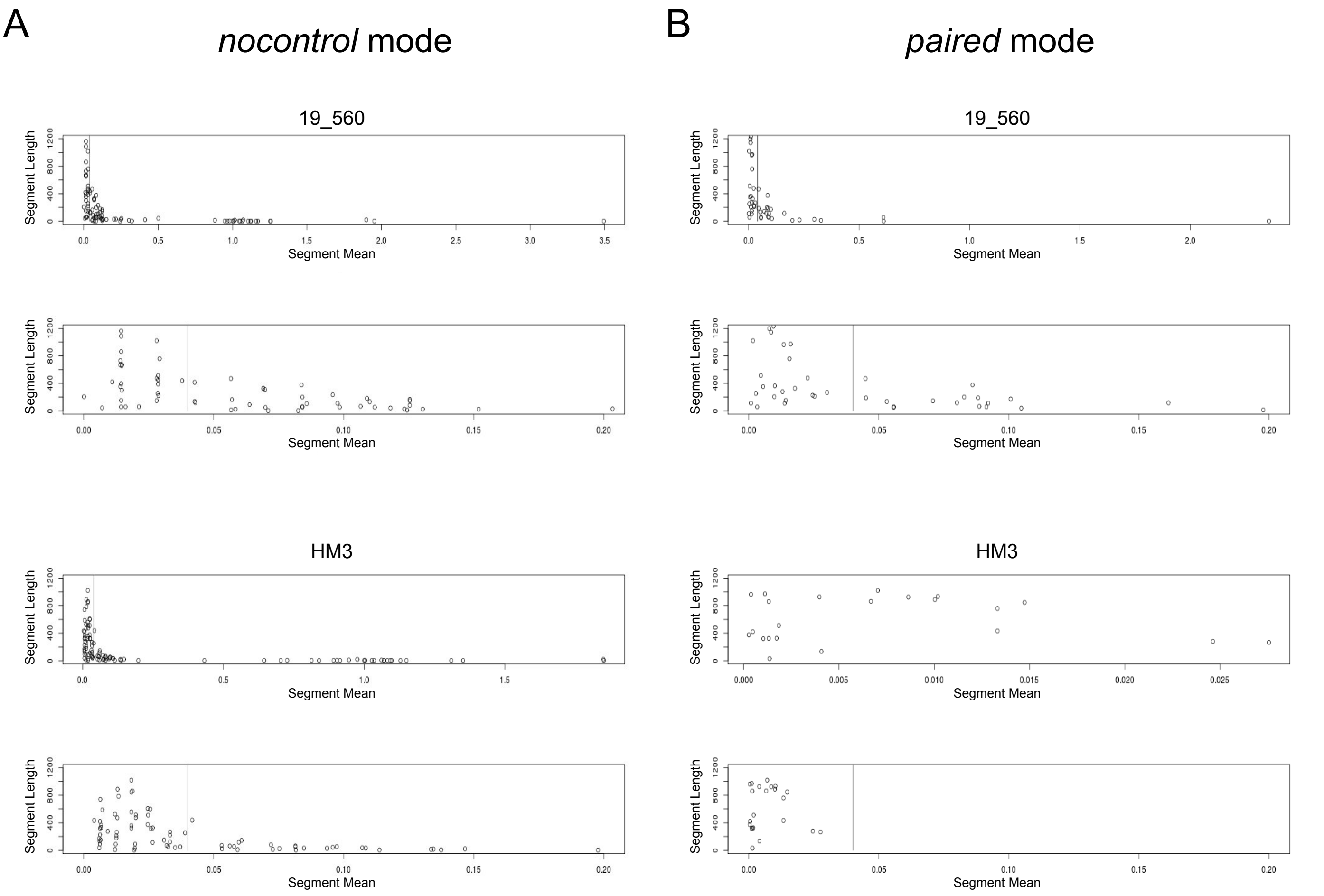

Figure S5. Segment mean and segment length of Nanopore results
Correlation of segment mean and length in nocontrol (A) and paired mode (B). Every dot represents a segment. Segment mean is reported on the x-axis and segment length (number of bins per segment) on the y axis. Vertical lines indicate the threshold used to discriminate artifacts from CNVs (log ratio ±0.04). The lower range of the segments is shown in the lower plot for each sample.

A

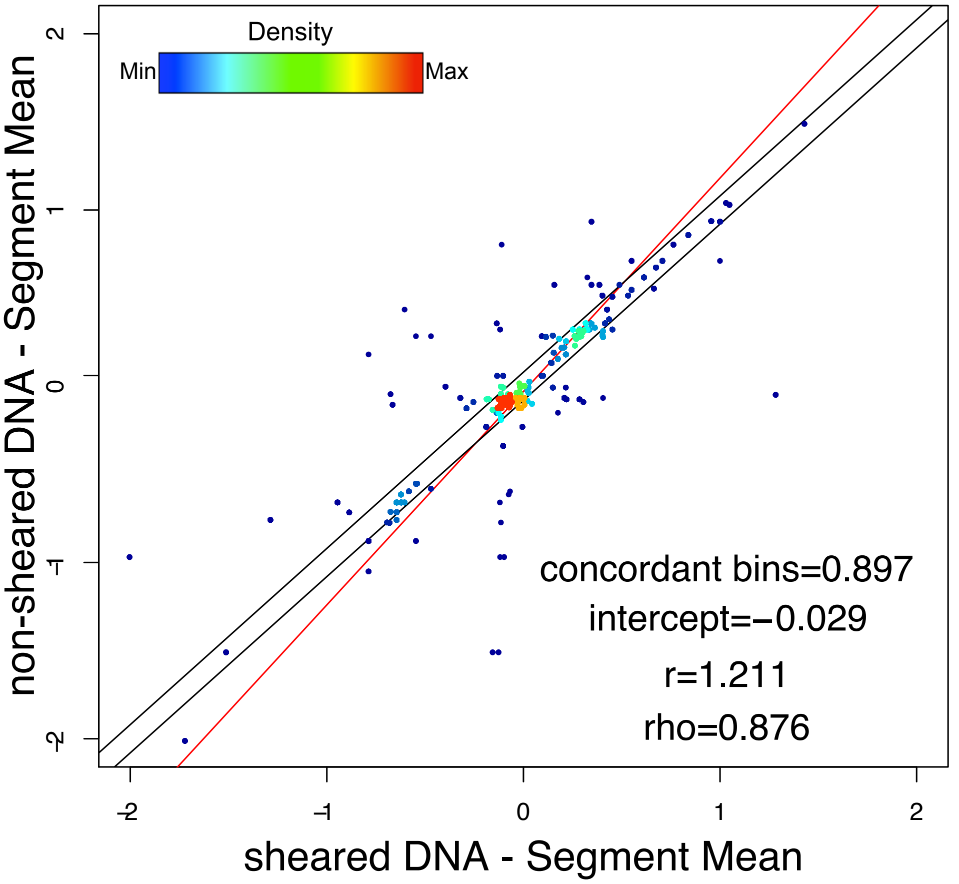

B

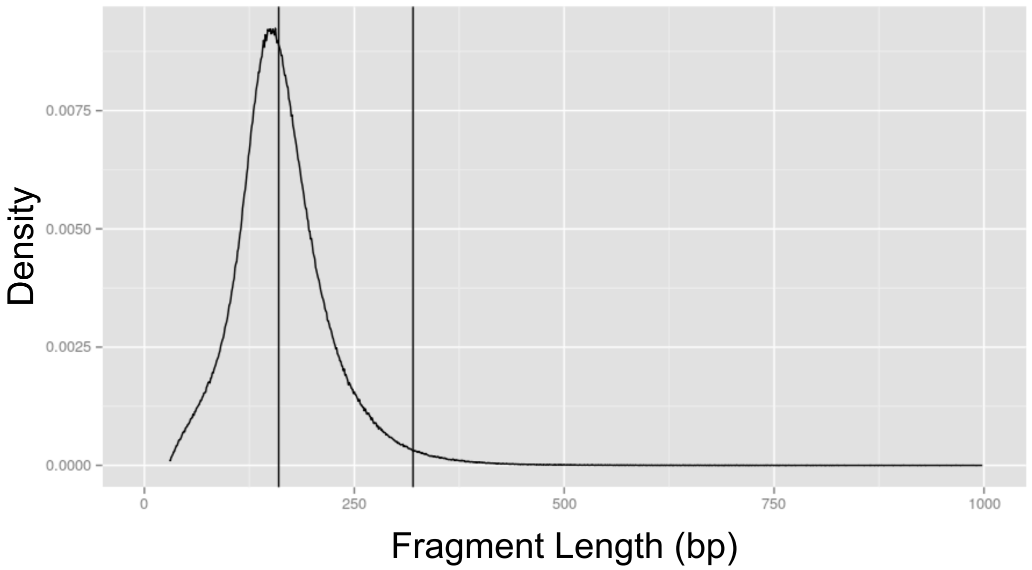

Figure S6. Correlation of short- and long-read sequencing results.
(A) Correlation plot of short-read (sheared DNA) and long-read (non-sheared DNA) sequencing. Each genomic bin is represented as a dot, colors indicate dot density. Regression lines are shown in red. Black lines indicate the thresholds for concordant bins.
(B) Fragment length distribution of HEK_sheared sample obtained from read length. Vertical lines indicates 160 and 320 bp length.

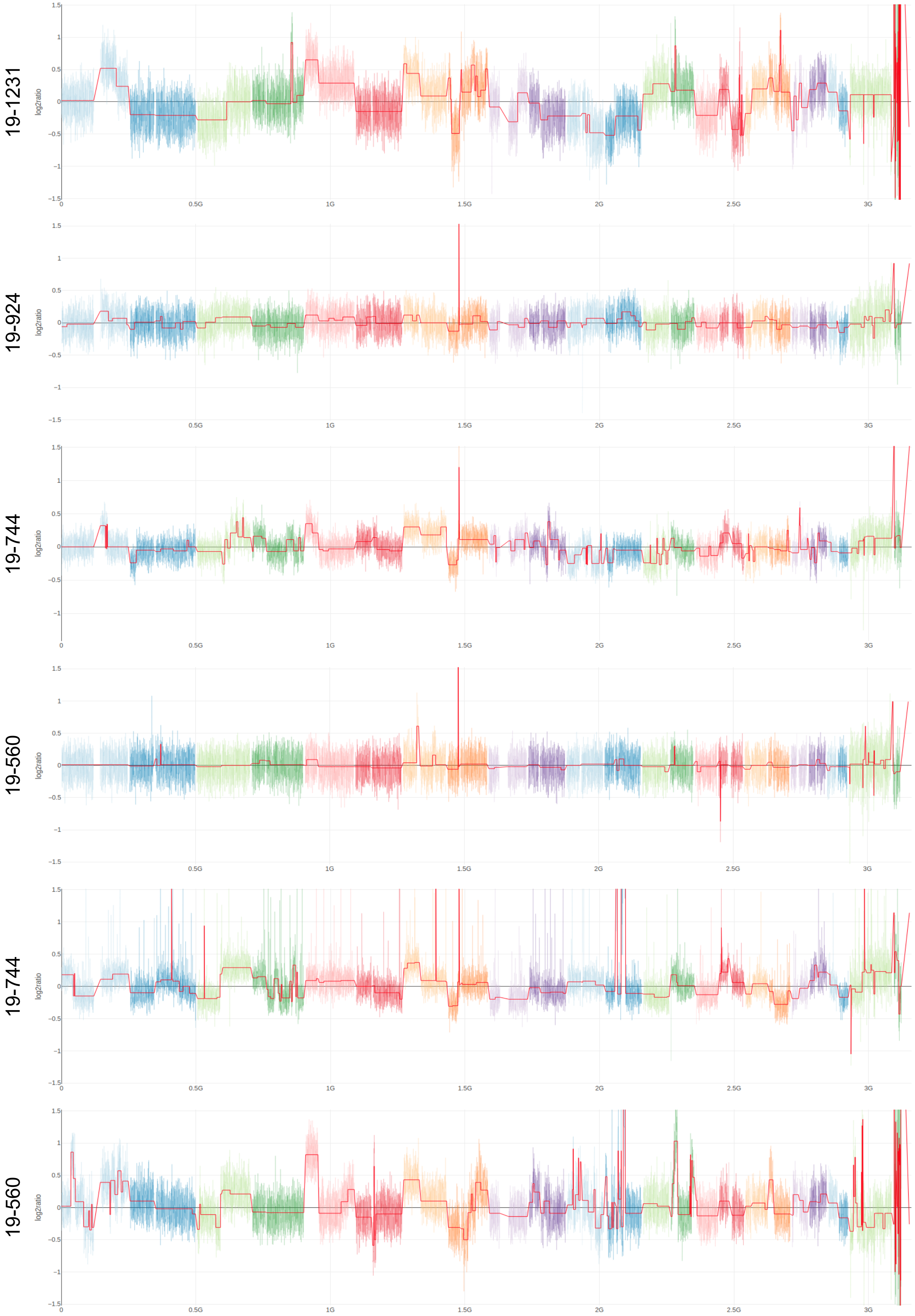

Figure S7. Segmentation results of cancer patients, *paired* mode
